# The influence of immediately loaded implant treatment in the atrophic edentulous maxilla on oral health related quality of life of edentulous patients: 3-year results of a prospective study

**DOI:** 10.1186/s13005-017-0154-0

**Published:** 2017-11-10

**Authors:** Maria Erkapers, Susanna Segerström, Karl Ekstrand, Russell A. Baer, Joseph A. Toljanic, Andreas Thor

**Affiliations:** 1 Department of Prosthetic Dentistry, Box 1813, SE-751 48 Uppsala, Sweden; 2 The University of Oslo, Inst. Klin. Odont, Protetikk Boks 1109, Blindern, 0317 Oslo, Norway; 3 University Associates in Dentistry, 222 N. LaSalle Street, Chicago, IL 60601 USA; 4 Midwestern University College of Dental Medicine-Illinois, 555 31st Street, Downers Grove, IL 60515 USA; 5 Institute of Surgical Sciences, Department of Plastic and Oral & Maxillofacial Surgery, Uppsala University, SE-751 85 Uppsala, Sweden

**Keywords:** Dental implants, Edentulous, Immediate loading, Health related quality of life

## Abstract

**Background:**

To evaluate oral health related quality of life (OHQoL) in edentulous patients treated with immediately loaded implants in the maxilla.

**Methods:**

Fifty-one edentulous patients in two centers received six maxillary implants each were loaded within 24 h with provisional restoration. Definitive restoration was delivered 20–24 weeks later. OHQoL was evaluated preoperatively with the Oral Health Impact Profile 49 questionnaire (OHIP-49) and on five subsequent occasions. OHIP-49 includes seven domains representing functional limitation, physical pain, psychological discomfort, physical disability, psychological disability, social disability, and handicap. A reduction in OHIP scores indicated an improved OHQoL.

**Results:**

Forty-five patients reached the three-year follow up. OHQoL improved after treatment. A plateau of OHQoL improvement was observed at 12 months after surgery. The seven domains improved at different pace, 12 weeks to 12 months after treatment. OHIP showed continuously low scores with no significant changes at consecutive visits 12 months to three years after treatment. Dental status with removable prosthesis in the mandible had a negative impact on OHQoL prior to and during treatment, but did not affect OHQoL after permanent restoration was placed. Patients age or gender did not affect OHQoL.

**Conclusions:**

Patients with edentulous maxilla who received prosthetic rehabilitation on immediately loaded implants experienced the highest improved OHQoL 12 months after implant installation. Quality of life related to oral health continued to be high after three years. Edentulous patients with atrophy of the maxilla experience an improved OHQoL after implant treatment with immediate loading protocol.

**Trial registration:**

ClinicalTrials.gov Identifier NCT00711022.

## Background

Rehabilitation with implant-retained prostheses is a well-recognized treatment when replacing missing teeth in edentulous jaws [1]. Treatment with complete dentures is less costly, but patient satisfaction/(OHQoL) is often lower compared to implant retained prosthesis. [2–9].

Dental implant rehabilitation has traditionally been provided as a two-stage surgical procedure with a conventional loading protocol. Implants have been placed submerged and unloaded for 12–24 weeks during their healing period [1]. The procedure with a conventional loading protocol is recognized and thoroughly documented. Improvement of implant surface technology has shortened the healing period from 12 to 24 weeks to 6–8 weeks. The technique with dental implants followed by early and immediate loading has emerged as an alternative treatment to the standard loading protocol [10]. Loading within 24–48 h after surgery is considered to be immediate. The procedure with immediately loaded implants, fixed as well as removable prostheses, may result in shorter treatment time, fewer surgical interventions and eliminates the need for a temporary removable prosthesis [11]. Promising results have been achieved with this treatment strategy [12, 13]. However, important parameters such as aesthetic outcome and patient OHQoL are often underexposed in implant studies [14]. The Oral Health Impact Profile 49 (OHIP-49) provides a recognized method to follow improvements and regression in OHQoL [15–18]. The OHIP measures frequency of oral problems and gives a profile of how affected a patient’s life can be because of these problems.

OHQoL for patients receiving fixed prostheses with conventional loading protocol versus immediately loaded implants in the edentulous maxilla is described with varying results [11, 19, 20]. Few long-term follow-up studies have been performed in this area.

The aim of the present study was to evaluate OHQoL before and after prosthetic rehabilitation with immediately loaded implants in atrophic maxillae using the OHIP-49 over a period of 3 years. The secondary aim was to investigate if prosthetic complications, status in the opposing dentition or the age or gender of the subjects would influence the OHQoL outcome. The null hypothesis was that OHQoL as reported in the OHIP-49 would increase after treatment regardless of prosthetic complications, status in the opposing dentition or subject age.

## Methods

The present study was performed in two centers.

Center 1: The Section of Dentistry, University of Chicago, Illinois, USA.

Center 2: The Department of Surgical Science, Oral Maxillofacial Surgery, Uppsala University, Sweden.

The Ethics-Committees of both institutions granted approval for the study.

### The Oral Health Impact Questionnaire (OHIP 49)

The OHIP-49 Questionnaire consists of 49 unique questions regarding patients’ oral health. The 49 questions can be divided into seven domains [18]. The seven domains describe different oral health impact problems (Table 1): There are five categories of choice per question: never, hardly ever, sometimes, fairly often or very often [4, 18, 21]. The categories are graded from 0 to 4, where 0 = never and 4 = very often. There is also an option to report if the question is not applicable. Higher OHIP scores indicate that a patient’s life is more affected due to a high frequency of oral problems resulting in a lower OHQoL. In this study OHIP scores were analyzed according to an ordinal scale. The internal reliability, test/retest reliability, and validity of the OHIP-49 have previously been established [16, 17]. A Swedish version of the OHIP-49 (OHIP-S) was used in the Swedish cohort. The OHIP-S has also been evaluated with reliability and validity tests [22]. OHIP-49 reveals both improvements and deterioration in patients’ health experience, enhancing the possibility to analyze change longitudinally [17].

**Table 1:** OHIP-49 divided in the seven domains, with example of statement for each domain

Domain	Questions	Example of statements
1 Functional limitation	1–9	I’ve had trouble pronouncing some words
2 Physical pain	10–18	My dentures have been uncomfortable
3 Psychological discomfort	19–23	I’ve been a bit irritable because of dental troubles
4 Physical disability	24–32	My speech has been unclear
5 Psychological disability	33–38	My concentration has been affected
6 Social disability	39–43	I’ve been less tolerant of my spouse
7 Handicap	44–49	I’ve been limited in my work

### Power analysis and subjects

A total of fifty-one patients were included in the trial. The size of the trial was not determined using statistical considerations but it was judged that 39 patients should be enough to determine clinically relevant changes in OHIP over time. A 10% difference in total OHIP scores was considered clinically relevant. Using the standard deviation for the percent decrease in the present trial (22.68) and a two-sided paired t-test, the statistical power to detect a difference of 10% is 88% when the number of patients is 51. If the number of patients is 45 the power is 84%.

Inclusion criteria were set as subjects of at least 20 years of age with a completely edentulous maxilla for at least 3 months. Clinical and radiographic findings had to indicate bone quantity types according to Lekholm and Zarb [23] C, D or E, and bone quality of 3 or 4 sites for proposed implant placement. In the mandible, dental support to the second premolars had to be present. This was accomplished with natural teeth, implants and prosthetic appliances (fixed partial dentures, removable partial dental prosthesis, implant supported prosthesis or overdentures). Patients were prosthetically rehabilitated in the mandible previous to entering the study.

Patients needed to provide informed written consent and return for all study visits as outlined in the study plan. Patients were excluded if they were unable to comply with all study procedures such as, if they had uncontrolled systemic or dental disease, if they had a history of chemotherapy or head and neck radiotherapy, alveolar bone augmentation surgery 6-month prior the study, or tobacco use 6-month prior the study, or if they were pregnant. A total of 51 patients scheduled for implant treatment in the edentulous atrophic maxilla were included and gave written consent to participate in the study. Of the 27 females and 24 male subjects, the age range was between 47 and 83 years, with a mean age of 66 years (Table 2). In all, 306 implants were placed in the maxilla of 51 subjects; 26 patients were treated at center 1 (USA) and 25 patients were treated at center 2 (Sweden). If several implants were lost, patients were offered additional second surgery or alternative solutions. Economic compensation was not offered for participation in the study.

**Table 2:** Characteristics of the study sample at baseline and differences between centers

Variable	Center 1	Center 2	Center 1 + 2	Significance
Gender				
Male	17	7	24	s^a^ 0.0135
Female	9	18	27	s^a^ 0.0135
Age (mean)	61.15	70.60	65.8	s^b^ 0.0002
Status opposing dentition				
Natural teeth	21	19	40	ns^c^
Fixed prosthesis	7	13	20	ns^c^
Removable prosthesis	16	10	26	ns^c^
Implant supported prosthodontics	5	3	8	ns^c^

*s*significant

*ns* non significant

^a^Fisher’s exact test. Two-sided *p*-value

^b^Wilcoxon rank sum test. Two-sided *p*-value

^c^Fisher’s exact test. Two-sided *p*-value

### Clinical protocol

Patients received six screw-shaped, self-tapping implants in the maxilla (OsseoSpeed**™** Dentsply Implants, Mölndal, Sweden). Implant surgery was performed under infiltrative local anesthesia using standard accepted flap tissue designs. A clear acrylic resin surgical stent, fabricated by duplicating the denture previously worn by the subject, was used to guide implant placement. The maximum possible antero-posterior spread of the implants was used in accordance with bone quality and quantity. No augmentation procedures were allowed [24].

The same surgical and prosthodontic protocol was utilized for all patients with the exception of the provisional fixed prosthesis technique. Center 1 used a direct, chair side technique for the provisional restorations in contrast to center 2 where an indirect technique was used. Both centers loaded the implants with a provisional fixed prosthesis within 24 h after surgery. The impression for the permanent restoration was made 12 weeks after implant placement on abutment level and the definitive restorations was placed 20 to 24 weeks after surgery. The definitive prosthetic construction was a screw retained metal-acrylic implant bridge.

Survival of the implants was assessed with clinical and radiologic examinations throughout the study as presented by Toljanic et al. [24].

The OHIP-49 was recorded and used to follow the change in general health experience associated with the oral health alteration. The participants were asked to complete the OHIP-49 questionnaire on six occasions combined with their clinical evaluation visits: prior to implant surgery and at 12 weeks, 6 months, 12 months, 24 months and 36 months after receiving the implants. The subjects completed the questionnaires at the study center by themselves, to prevent being influenced by other individuals.

### Statistical analysis

The change over time in OHIP was analyzed using a non-parametric approach as the data are ordered categorically and hence the normality distribution can be questioned. To compare the centers, the Wilcoxon rank sum test was used for continuous variables and the Fisher exact test was used for categorical variables. The null hypothesis – that the change over time in OHIP-49 scores would be equal to zero – was tested by means of the Wilcoxon signed rank test. The hypothesis that the percent change over time would be equal at the centers was also tested by the means of Wilcoxon signed rank test. All reported *p*-values were two-sided. Statistical significance was considered when *p* < 0.05. If OHIP values were missing they were imputed using the mean of the non-missing values for that patient in the respective cluster. The definition of missing OHIP value was; Question not answered, or patients reported that the question was not applicable. No adjustment for multiplicity was conducted since the risk for multiplicity should be taken into account in the clinical conclusion (but as the results showed no statistically significant difference this was not applicable). Pooled data from the two centers were evaluated, and a comparison between centers was performed. Total OHIP-49 scores were followed over time, along with individual data from the seven different domains. The number of prosthetic repairs of the final implant supported prosthesis was analyzed regarding the possible impact on the OHIP results. The correlation between OHIP scores and variables, such as age, gender and status of the dentition in the mandible, was evaluated by comparing OHIP total and each of the subgroups (1–7). The OHIP scores were compared at each time of registration. Changes between consecutive visits were also compared. A reduction in OHIP scores indicated an improved OHQoL.

## Results

### Participants

Baseline characteristics for the participants are shown in Table 2. Statistical significant differences between the two centers were identified regarding gender and age, but no statistically significant differences were identified regarding the dental status in the mandible. Six patients were excluded. Three patients were excluded due to loss of implants. After three years, 13 implants had been lost (three implants in three patients at center 1 and ten implants in three patients in center 2). Two patients discontinued the study, one patient was lost to follow-up, and one patient was not willing to continue the study. One patient was deceased. The OHIP scores for these patients were therefore not included in the analysis. Forty-five patients reached the three-year follow up.

### OHIP

**Fig. 1:**
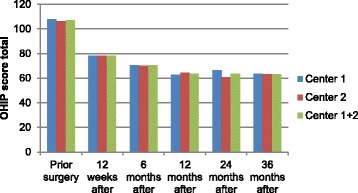
Total OHIP scores over 3 years. Change in mean values for total OHIP scores over time at six different occasions for each centre and for both centers combined

**Fig. 2:**
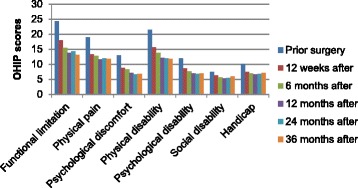
The seven domains over 3 years. Mean OHIP scores on six occasions for each domain for the both centers represented as one group

A total of 368 OHIP values were missing (65 at visit 1, 78 at visit 5, 38 at visit 7, 26 at visit 8, 40 at visit 9, 40 at visit 10, 44 at visit 11 and 37 at visit 12) of a total of 17,738, i.e. 2%. Missing OHIP values were imputed using the mean of the non-missing values for that patient in the respectively cluster. The risk for multiplicity should be taken into account in the clinical conclusion, but as the results showed no statistically significant difference this was not applicable. Hence no adjustment for multiplicity was conducted. Baseline OHIP scores before treatments were similar for the two research centers. Overall satisfaction after treatment was significantly improved for both centers as shown in Fig. 1. All seven domains demonstrated lower OHIP scores after treatment with no statistical significant difference between centers, as displayed in Fig. 2. The first five domains (functional limitation, physical pain, psychological discomfort, physical disability, and psychological disability) showed greater improvement compared to domain six and seven (social disability and handicap).

The total OHIP score in the seven domains between baseline and between each consecutive visit were evaluated as change in OHQoL.

The pace of improvement was different for the seven domains (Table 3). Continuous OHIP improvement for the seven domains was recorded twelve weeks to twelve months after implant surgery. Twelve weeks after surgery domains two, three, five, six and seven showed stagnations in improvement. OHIP total as well as, domain one and four showed a plateau in improvement twelve months after treatment. After two years, a minor, non-significant increase in OHIP scores for domain one and two were noticed. After three years, a minor, non-significant increase in OHIP scores for domain three, six and seven were noticed.

**Table 3:** OHIP-improvement (expressed with *p*-values) between consecutive visits for the domains and OHIP total

OHIP-domains	Prior surgery-12 weeks’ after	12 weeks to 6 months’ after	6–12 months’ after	12–24 months’ after	24–36 months’ after
OHIP total	s 0.0000	s 0.0320	s 0.0046	ns	ns
Domain 1	s 0.0000	s 0.0127	s 0.0001	ns	ns
Domain 2	s 0.0000	ns	ns	ns	ns
Domain 3	s 0.0001	ns	s 0.0491	ns	ns
Domain 4	s 0.0000	s 0.0438	s 0.0062	ns	ns
Domain 5	s 0.0001	ns	ns	ns	ns
Domain 6	ns	ns	ns	ns	ns
Domain 7	s 0.0000	ns	ns	ns	ns

*s* significant

*ns* non-significant

*p*-value >0,05

Thirteen participants (29%) had one or more prosthetic complication after three years. Table 4 lists the different prosthetic complications recorded. Patients experiencing prosthetic complications did not show different results in the seven domains or OHIP-total improvement compared to trouble-free patients at the three-year follow-up.

**Table 4:** Prosthodontic complications after three years

Prosthodontic complications	Numbers
Fractured denture tooth	15
Inaccurate seating of angled titanium cylinder	2
Fractured resin provisional bridge	1
Excessive occlusal contacts	1
Food impaction	2
Framework fracture	3
Abutment fracture	1
Abutment loose	2
Phonetic problems	1
Irregularities	1
Bridge screw loosening	3
Construction too bulky	1

No differences in OHQoL were evident between patients <60 and >60 years or between gender.

Status of the mandible was sub-grouped (natural teeth, fixed prosthesis, removable prosthesis or implant retained prosthodontics) and compared. Patients with removable prostheses recorded the lowest OHQoL prior and after implant installation during the use of the provisional prosthetic construction in the maxilla. Patients with implant-retained prosthodontics in the lower jaw recorded the highest OHQoL prior treatment and the least improvement after treatment compared to other groups. All groups showed equally high OHQoL/low OHIP scores after permanent restoration was placed.

## Discussion

The OHIP-49 questionnaire used in this study provides a well-established method to measure OHQoL change [18]. OHIP-49 is the original questionnaire; however shorter modified versions exist ranging from 5 to 30 queries. OHIP-EDENT is a shorter modified version of OHIP-49 questionnaire that could be better applicable and for use in edentulous patients. However, OHIP-EDENT has not been evaluated with reliability and validity tests in the Swedish population. Also, a shorter version is less time-consuming but does not show the same accuracy as the complete OHIP-49 version does [18, 21, 25]. Therefore, OHIP-49 was used in this study. To minimize the risk of not completing the questionnaire or being influenced by friends and family, the participants were asked to complete the form at the centers when they were called in for their routine examinations. OHIP-49 questionnaire contains oral health questions. Some of the questions might not be valid for all patients after completed implant treatment, especially if they were not denture wearers. For example, questions: 9) *Have you felt that your dentures have not been fitting properly?*18) *Have you had uncomfortable dentures?* 30) *Have you been unable to eat with your dentures because of problems with them?*

Patients have an option to say that a question is not applicable, but inessential questions might cause confusion. This might be worth further reflection depending on which type of treatment one aims to evaluate with OHIP-49.

In the present multicenter study, no differences were found between the centers when comparing changes in OHQoL before and after treatment. Therefore, the two centers could be analyzed as one group, in spite of the lack of random allocation to the centers. A two-center study also has the benefit of being able to treat more patients than one center alone can treat in a relatively short time. In this study, it would have been beneficial with a control group with a two-stage surgical procedure and a conventional procedure.

A two-stage surgical procedure involves periods of healing before loading. During this healing period, patients usually wear a removable denture. In this study, included patients had severely resorbed maxillae resulting in unfavorable/impossible conditions for a removable denture, therefore it was impractical with a control group.

The two-stage surgical protocol is a well-documented procedure with high survival and success rate of the implants [26, 27]. Improved patient satisfaction after dental implant treatment with two-stage surgical procedure and a conventional loading protocol has been established in earlier studies [3–5, 8, 28]. A conventional loading protocol may seem optional, but some issues should be addressed. Two surgical procedures are necessary with a conventional protocol. Patients are usually asked not to use their denture the first two weeks after the initial operation. This can be socially difficult or impossible for some patients [29]. A temporary removable denture during the healing period can be challenging for some patients. The technique with immediately loaded implants provides the patient with temporary fixed teeth in conjunction with the surgical intervention. Patients also have the opportunity to have a preview of what the permanent restoration can look like. This might be an advantage before delivering the final restoration, aiming for successful esthetic outcome and high patient satisfaction. However, the technique involved with immediate loading is demanding regarding the technical procedure by which the temporary fixed denture is manufactured. The procedure requires a competent dental technician and a skilled dentist since the stability of the temporary fixed denture may influence the prognosis of the implants.

The improvement of OHQoL (Oral Health-related Quality-of-Life) after treatment with immediately loaded implants is verified in this study. Immediate implant loading reduces the treatment period and the need for provisional removable dentures during the healing process, this might give a positive psychological advantage compared with conventionally loaded implants. This is in agreement with Eliasson et al. [30], who also concluded that patients treated according to the early loading concept are more satisfied than those treated according to conventional loading. In this study, an early plateau of improvement was reached twelve weeks after surgery for domains two, three, five, six and seven. For domain one and four the improvement leveled out twelve months after treatment. OHIP total/OHQoL reach a plateau of improvement twelve months after treatment. These results are comparable with those of Cannizzaro et al. [19], who concluded that patients with immediately loaded implants were significantly more satisfied compared to patients receiving conventionally loaded implants. Fischer and Stenberg [20] however found no difference in satisfaction between the groups. Penarrocha-Oltra et al. [11] found an earlier increased satisfaction for patients with immediate loaded implants compared to patients with conventional loaded protocol, but no difference was found 12 month after surgery. In this study, continuous positive steady level of OHQoL were found three years after surgery.

An early plateau of OHIP improvement (lowered scores) might be an indication that the highest level of improvement possibility has been reached. These results indicate, that treatment with immediately loaded dental implants generate the highest improvement for the domains between 12 weeks to 12 months after treatment, which is in agreement with previous studies [11, 19, 31, 32]. It is possible that some domains improved within 24 h after surgery when the implants were loaded with the provisional restorations, however no OHIP evaluation was made previous to 12 weeks. Domain one and four showed a delayed leveling of satisfaction twelve months after treatment. Domain one “functional limitation” and domain four “physical limitation” includes questions concerning the capacity of speech, chewing, and taste. It is possible that the delayed improvement in those domains is due to the fact that they include skills that has a longer improvement process compared to the other domains.

According to Eliasson et al. [30], immediately loaded implants have more prosthetic complications compared to conventionally loaded implants. Prosthetic setbacks were suspected to influence patients’ contentment. In this study, patients with prosthetic complications did not experience a lower quality of life related to oral health compared to patients presenting no prosthetic problems. Katsoulis et al. [33] received similar results when comparing treatment outcome of partially edentulous patients with severe tooth wear. A possible explanation may be that patients were informed regarding that prosthetic problems are commonly encountered and can be expected. The most common prosthetic failure reported was “fractured denture tooth”. A fractured denture tooth can easily be repaired and should be considered a complication and not a failure [34]. Framework fracture occurred three times on definitive restorations on two patients, one from each study center. One fractured on a patient from center one. Two fractures occurred on the same patient from center two. The two fractures arised from the same position of the framework twice, indicating an impairment of the framework. An implant-supported prosthesis needs maintenance and care. The information regarding advantages and disadvantages before and after dental implant treatment must therefore be considered as an important part of the treatment itself [35]. A well-informed patient, who accepts the risks with treatment, ought to be more qualified to cope with any prosthetic complications.

Age does not seem to have an impact on patient satisfaction for patients receiving dental implant treatment [36, 37]. No difference in OHIP outcome was noticed when comparing patients older or younger than 60 years of age, as also stated in the one year results by Furuyama et al. [36, 37]. However the opposite has also been reported [38].

Furthermore, our results coincide with earlier studies stating that oral health improvement after dental implant treatment was similar for males and females [38].

Patients’ status in the lower jaw was classified as follows; natural teeth, fixed prosthesis, implant retained prosthodontics or removable prostheses. Patients using removable prostheses experienced lower OHQoL prior and after implant installation during the use of the provisional prosthetic construction in the maxilla, but experienced equally OHQoL after receiving the permanent implant restoration. Thus, patients with removable prosthesis in the lower jaw are more affected of a provisional restoration in the maxilla comfort vice compared when a fixed dentition (natural teeth or implant retained teeth) is present in the mandible. OHQoL increased with the delivery of the permanent fixed implant construction. The increased OHQoL may be a result of an adaption period after the surgery, indicating that the patient is fully adapted to the implant supported prosthesis opposing the removable denture. This suggests that patients wearing a removable prosthesis in the mandible found the prosthesis to be functionally acceptable in combination with the implant-retained maxillary prosthesis and thus achieving a satisfying treatment result. Patients having implant retained prosthodontics in the mandible experienced the highest OHQoL prior to treatment, indicating that a stabile dental situation in the lower jaw increase acceptance for a less stable dental situation in the maxilla. After treatment, patients had equally low OHIP scores/ high OHQoL independent of status in the lower jaw. This result indicates that edentulous patients with severe atrophy of the maxilla will have a high OHQoL after implant treatment with permanent restoration independent of the status in the mandible.

OHIP domains one to five (functional limitation, physical pain, psychological discomfort, physical disability, and psychological disability) showed more improvement than domains six and seven (social disability and handicap). Domains six and seven had the lowest OHIP scores prior to treatment, indicating minor trauma from wearing a denture and making it more difficult to improve after treatment. This could be an explanation to the lower pace of change in OHIP score. The observation concerning less frequently reported consequences for social disability and handicap during implant treatment is supported in a previous study [15]. Domain three (psychological discomfort) showed more improvement than domain five (psychological disability). Domain three had lower OHIP scores prior treatment compared to domain five, indicating that patients were less affected by psychological discomfort compared to psychological disability and making it more difficult to improve. Our results suggest that both edentulous and dentate implant patients grade domains one to five (functional limitation, physical pain, psychological discomfort, physical disability, and psychological disability) to be more important for oral health comfort compared to domains six and seven (social disability and handicap). Another explanation to the larger change in the first domains might be that the mean OHIP score was higher in domain 1–5 compared to domains 6 and 7 before surgery, and one cannot improve what is already perfect. Therefore, the greatest improvement can be expected within domain 1–5 (functional limitation, physical pain, psychological discomfort, physical disability, and psychological disability). This finding could be an important guideline for the treating dentist. OHIP provides a patient perspective on the outcome of treatment of oral disorders and may help to evaluate the benefits of therapeutic measures, such as immediate loading on dental implants and prosthetic rehabilitation.

## Conclusion

OHQoL improved after prosthetic rehabilitation with immediately loaded implants in patients with edentulous atrophic maxillae, and remained three years after treatment. The null hypothesis was confirmed in this study. Quality of life related to oral health increased regardless of prosthetic complications, status in the opposing dentition or subject’s age. These results indicate that patients with edentulous atrophic maxillae can benefit from immediately loaded implant technique.

## Abbreviations

OHIP-49: Oral Health Impact Profile 49 questionnaire
OHQoL: Oral health related quality of life
